# Restless Head Syndrome and Burning Mouth Syndrome: Clinical Overlap and Diagnostic Pitfalls in Orofacial Pain

**DOI:** 10.3390/jcm15218576

**Published:** 2026-11-03

**Authors:** Katharina Theresa Obermeier, Anusha Abdullah, Felix Kramer, Ina Dewenter, Wenko Smolka, Michael Roßkopf

**Affiliations:** 1 Department of Oral and Maxillofacial Surgery and Facial Plastic Surgery, LMU University Hospital, Ludwig-Maximilians-Universität München, Lindwurmstraße 2a, 80337 Munich, Germany; 2 Department of Psychiatry and Psychotherapy, University of Regensburg, 93053 Regensburg, Germany

**Keywords:** restless head syndrome, restless leg syndrome, PLM, restless syndrome, dopamine agonist, polysomnography, restless mouth syndrome, restless face syndrome, burning mouth syndrome, orofacial pain

## Abstract

Restless Head Syndrome (RHS) and Burning Mouth Syndrome (BMS) are rare, underrecognized causes of orofacial discomfort that are frequently misdiagnosed. This review maps the available literature on both conditions, examines their proposed relationship to restless leg syndrome (RLS) and to each other, and appraises the role of dopaminergic therapy. A literature search was conducted in PubMed and ScienceDirect for publications from January 2000 to December 2024 describing the clinical presentation, diagnosis, or treatment of RHS, BMS, or craniofacial variants of RLS. The evidence consisted predominantly of case reports and small case series. RHS and other craniofacial RLS variants presented with rest-associated, evening-predominant discomfort that was relieved by movement and responded to dopaminergic agents. Only one small case series described patients with BMS who also fulfilled RLS criteria. The evidence supports clinical similarities rather than a confirmed association or shared pathophysiology. Clinicians should consider atypical RLS variants in the differential diagnosis of unexplained orofacial sensory symptoms; prospective studies are needed before firm diagnostic or therapeutic conclusions can be drawn.

## 1. Introduction

Patients with orofacial pain and neuropathic pain of the face are a special patient clientele, often difficult to treat [1]. Chronic orofacial pain is generally defined as pain occurring on at least 15 days per month, although a clear definition of orofacial pain does not exist [1]. According to the International Association for the Study of Pain (IASP), orofacial pain can be defined as “an unpleasant sensory and emotional experience associated with actual or potential tissue damage or described in terms of such damage” [2]. Orofacial pain has a multifactorial etiology that can be either odontogenic or non-odontogenic. It can be caused by functional disorders, structural abnormalities, as well as psychological factors [3]. More specifically, odontogenic causes include dental caries, pulpitis, apical periodontitis, periodontal disease, and post-procedural complications. Musculoskeletal pain commonly arises from myofascial dysfunction of the masticatory muscles and temporomandibular disorders involving joint inflammation, degenerative changes, or internal derangement. Neuropathic conditions include trigeminal neuralgia and painful trigeminal neuropathy, whereas persistent idiopathic facial pain and burning mouth syndrome may occur without clinically identifiable tissue pathology. Peripheral nociceptive input, altered central pain processing, and psychosocial factors may interact to influence symptom persistence and severity [1,4,5]. This condition may lead to depression and sleeping disorders, finding the trigger for the disease is often difficult [3,4].

Orofacial pain is common worldwide. A recent meta-analysis of 155 observational studies estimated a global prevalence of 21% for oral pain, 12% for facial pain and 19% for concurrent oral and facial pain [6]. Among orofacial pain subtypes, pain attributed to disorders of dentoalveolar and anatomically related structures is the most frequent (27%), followed by myofascial orofacial pain (21%), combined myofascial and temporomandibular joint (TMJ) pain (12%) and TMJ pain alone (10%), whereas orofacial pain attributed to lesions or diseases of the cranial nerves is reported in 8% [7]. Both analyses showed very high between-study heterogeneity, so these estimates should be interpreted with caution [6,7]. These pooled estimates refer to different clinical categories and study populations; they are not mutually exclusive and should not be added together. The distinction between common odontogenic or temporomandibular causes and less frequent neuropathic or idiopathic presentations is particularly relevant when patients report persistent pain or dysesthesia despite unremarkable dental and mucosal examinations [1,5,7]. Against this background of a high overall burden, conditions without a structural correlate, such as BMS and craniofacial variants of restless leg syndrome, represent a small but clinically important subgroup in which diagnostic uncertainty is greatest.

While many of these conditions can be caused by TMJ disorders and trigeminal neuralgia, there are certain less common and underdiagnosed syndromes that should be considered as potential differential diagnosis [8]. Among those are restless head syndrome (RHS) and burning mouth syndrome (BMS), which share characteristics such as chronic pain, nervous dysfunction and psychological impact [9]. Recognizing these disorders is particularly important in patients presenting with pain in the head and neck area and the oral cavity without identifiable clinical findings [10].

RHS is known as a rare subtype of restless leg syndrome (RLS). RLS is a neurological disorder characterized by an irresistible urge to move the legs accompanied by uncomfortable sensations like throbbing, pulling and creeping [11,12]. Symptoms normally occur in the evening hours and may cause sleeping disturbances. RLS is associated with reduced quality of life and, in severe cases, depression and suicidal thoughts [13]. The prevalence of RLS is between 9–15% depending on the study [14]. The pathogenesis and causes can be diverse. The idiopathic or primary form can be genetic. The secondary form can be caused by iron deficiency and drugs like neuroleptics and antidepressants. Dopamine seems to play an important role as agonists have a positive therapeutic impact [15]. Diagnosis is primarily clinical and based on the criteria defined by the International Restless Legs Syndrome Study Group (IRLSSG) [11]. Although RLS predominantly affects the lower extremities, variants involving the arms (Restless Arm Syndrome, RAS), abdomen, genitalia, bladder, lower back and the craniofacial region have been described [16,17,18].

RHS is a condition characterized by an incessant feeling of discomfort or the urge to move the head [19]. While the exact prevalence of RHS remains uncertain, it is suggested to be more common than previously assumed. Symptoms resemble those of RLS and may also impair sleep and daily functioning [12]. Only a few cases of RLS involving the face have been reported in the literature [20]. For example, Prakash et al. (2020) described two patients with RLS who also experienced chronic tension-type headaches, both of which responded positively to treatment with levodopa [21]. The authors proposed this as a cephalic variant of RLS, in which the headache component shows a significant response to dopaminergic therapy.

The terminology of craniofacial RLS variants is not uniform, and three overlapping descriptive terms are used in the literature: (i) restless head syndrome, referring to discomfort or an urge to move localized to the head, scalp or neck, sometimes presenting as a dopa-responsive headache [19,21]; (ii) restless face, referring to facial paraesthesias or dysaesthesias with RLS characteristics [20,22,23]; and (iii) restless mouth or the oral variant of RLS, referring to intraoral discomfort, including burning, of the tongue, jaw or oral mucosa [18,24,25]. These presentations may occur in isolation or together with classical limb symptoms and are regarded as atypical, extra-limb manifestations of RLS rather than separate disease entities [17]. None of them has validated, site-specific diagnostic criteria; published cases were diagnosed by applying the essential IRLSSG criteria (an urge to move accompanied by unpleasant sensations, onset or worsening during rest, at least partial relief by movement, evening or night-time predominance, and symptoms not solely accounted for by another condition) to the craniofacial region [11,17]. In this review, “craniofacial RLS variants” is used as the umbrella term, whereas RHS, restless face and restless mouth refer only to the specific locations described above. Of these, the restless mouth (oral) variant overlaps most closely with BMS.

BMS, in contrast, is defined in the International Classification of Orofacial Pain 2020 as “Idiopathic orofacial pain with intraoral burning or dysesthesia recurring daily for more than 2 h per day and more than 3 months, without any identifiable causative lesions, with or without somatosensory changes” [5]. Primary BMS can be found between 0.11–3.7% of the population, prevalence in women is higher than in men [26]. Secondary BMS is associated with underlying local or systemic pathological conditions, including vitamin B deficiencies, medications, infections, and other medical disorders [27]. Patients frequently report burning sensations, allodynia, electrical discharges, numbness and prickling pain, which may persist or fluctuate in intensity throughout the day. However, diurnal pain may also appear [28,29].

The rationale for considering RHS and BMS together is primarily clinical rather than mechanistic. Both are diagnoses of exclusion that present with chronic, lesion-free sensory symptoms of the craniofacial region; both lack objective diagnostic markers; and both are associated with sleep disturbance and psychological comorbidity [9,12,30]. Most importantly, an oral variant of RLS can fulfil the ICOP definition of BMS: in a case series of patients with dopa-responsive BMS, four of five also met the IRLSSG criteria for RLS [24]. A patient with a craniofacial RLS variant may therefore be classified as having BMS, and vice versa, with direct consequences for treatment. The aim of this review was therefore to map the available literature on RHS, other craniofacial RLS variants and BMS, to describe their clinical similarities and distinguishing features, and to critically appraise the evidence for a proposed association and for dopaminergic therapy, rather than to establish a shared pathophysiology.

## 2. Material and Methods

A literature search was conducted in PubMed and ScienceDirect to identify publications related to Restless Head Syndrome (RHS) and Burning Mouth Syndrome (BMS). The search was performed for publications between January 2000 and December 2024. Reporting followed the PRISMA extension for scoping reviews (PRISMA-ScR) where applicable [31].

The following search terms and combinations were used:

(“restless head syndrome” OR “head restlessness” OR “periodic head movements” OR “dental pain”) AND (“orofacial pain” OR “craniofacial pain” OR “burning mouth syndrome”).

These terms were chosen to capture RHS and related head restlessness, orofacial pain conditions with which RHS may be confused, including dental pain, and BMS.

### 2.1. Inclusion Criteria

- Articles published in English;
- Human studies (case reports, case series, observational studies, clinical trials, reviews);
- Studies describing clinical presentation, diagnosis, or treatment of RHS, BMS, or RLS variants affecting the head, face, or oral region;
- Publications between 2000–2024; earlier publications identified through backward citation searching were additionally included if they were original case descriptions of craniofacial RLS variants.

### 2.2. Exclusion Criteria

- Animal studies;
- Articles without clinical relevance to RHS, BMS, or RLS variants;
- Studies lacking sufficient clinical or diagnostic detail;
- Duplicate publications.

### 2.3. Study Selection

**Figure 1:**
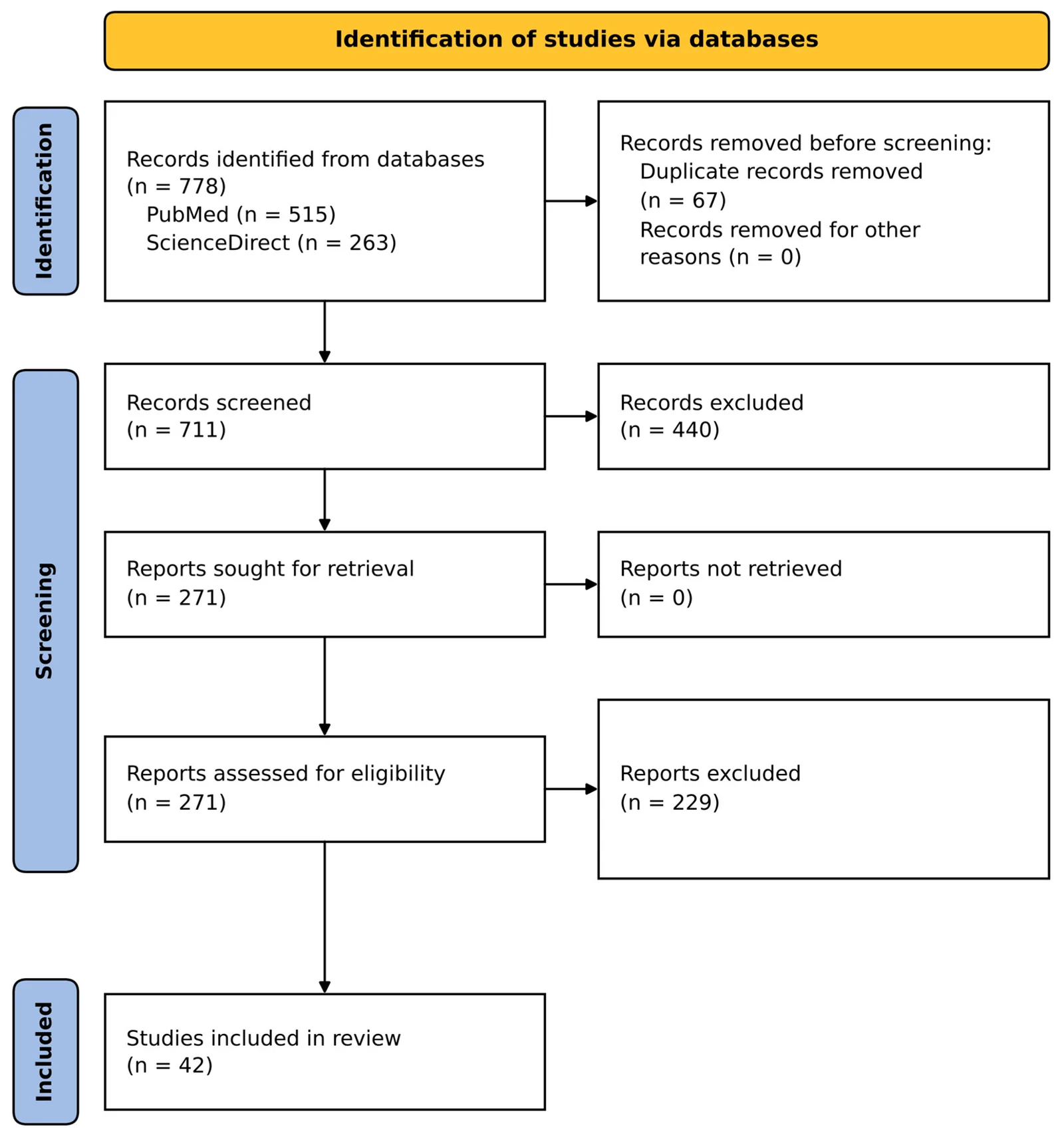
PRISMA flow diagram of the literature search and study selection.

All references retrieved from the searches were imported into EndNote, and duplicates were removed. Titles and abstracts were screened independently by two reviewers to assess eligibility. Full-text articles were then reviewed to determine final inclusion. Disagreements were resolved through discussion until consensus was reached. The number of records identified, screened and excluded and the number of publications finally included are reported in a PRISMA flow diagram (Figure 1).

### 2.4. Data Extraction

Data extracted from each included study comprised: authors, year of publication, sample size, clinical presentation, diagnostic approach, treatment, and reported outcomes. Due to the heterogeneity and limited number of published cases, a meta-analysis could not be performed. Therefore, results were synthesized narratively. To separate direct from indirect evidence for an association, the publications summarized in Table 1 were grouped into (A) RHS and other craniofacial RLS variants without BMS, (B) BMS with concomitant RLS features, and (C) BMS without RLS features. Given that the. Available literature consistes almost exclusively of case reports and small case series, no formal scored critical appraisal was performed, in line with PRISMA-ScR; instead, studies were characterized by design and by the type of evidence they provide.

**Table 1:** Selected overview of clinical characteristics and treatment outcomes reported in case reports and case series describing RHS, BMS, and craniofacial variants of RLS.

Author(s), Year	Study Design (n)	Clinical Presentation	Reported RLS Diagnostic Features	Treatment and Outcome
***Group A: RHS and other craniofacial RLS variants (indirect evidence regarding BMS)***				
Fukunishi et al., 1998 [23] *	Case report (n = 1)	Facial paresthesia in a patient undergoing hemodialysis	Symptoms worsened at rest and in the evening and improved with movement	Resolution of symptoms with levodopa
Buchfuhrer, 2008 [20]	Two case reports (n = 2)	RLS with extension of symptoms to the face	Classical RLS reported	Reduction in symptom severity with ropinirole
Jung et al., 2017 [25]	Case report (n = 1)	Oral discomfort at rest accompanied by an urge to move the jaw	RLS-like features reported; complete criteria not specified in the table	Symptomatic improvement with pramipexole
Suzuki et al., 2017 [22]	Case report (n = 1)	Abnormal facial sensations consistent with restless face	All IRLSSG criteria reportedly fulfilled	Complete resolution with pramipexole (0.125 mg/day)
Wolters and van Nuenen, 2020 [18]	Case reports (n = 2)	RLS involving the upper limbs and tongue	RLS diagnosis reported; individual criteria not specified in the table	Symptom control with pramipexole
Prakash and Prakash, 2020 [21]	Case reports (n = 2)	Cephalic RLS variant associated with chronic tension-type headache	Limb RLS reported	Reduction in headache symptoms with levodopa
***Group B: BMS with concomitant RLS features (direct evidence of clinical overlap)***				
Prakash et al., 2012 [24]	Case series (n = 5)	Burning mouth symptoms responsive to dopaminergic treatment	RLS features reported in four of five patients	Improvement with levodopa/carbidopa; recurrence following dose reduction or discontinuation
***Group C: BMS without RLS features (comparison of symptom pattern)***				
Forssell et al., 2012 [29]	Prospective pain-diary study (n = 52)	Primary BMS	Not assessed	No intervention evaluated; intermittent symptoms and mean pain intensity of 3.1 ± 1.7 (NRS)

**Figure 2:**
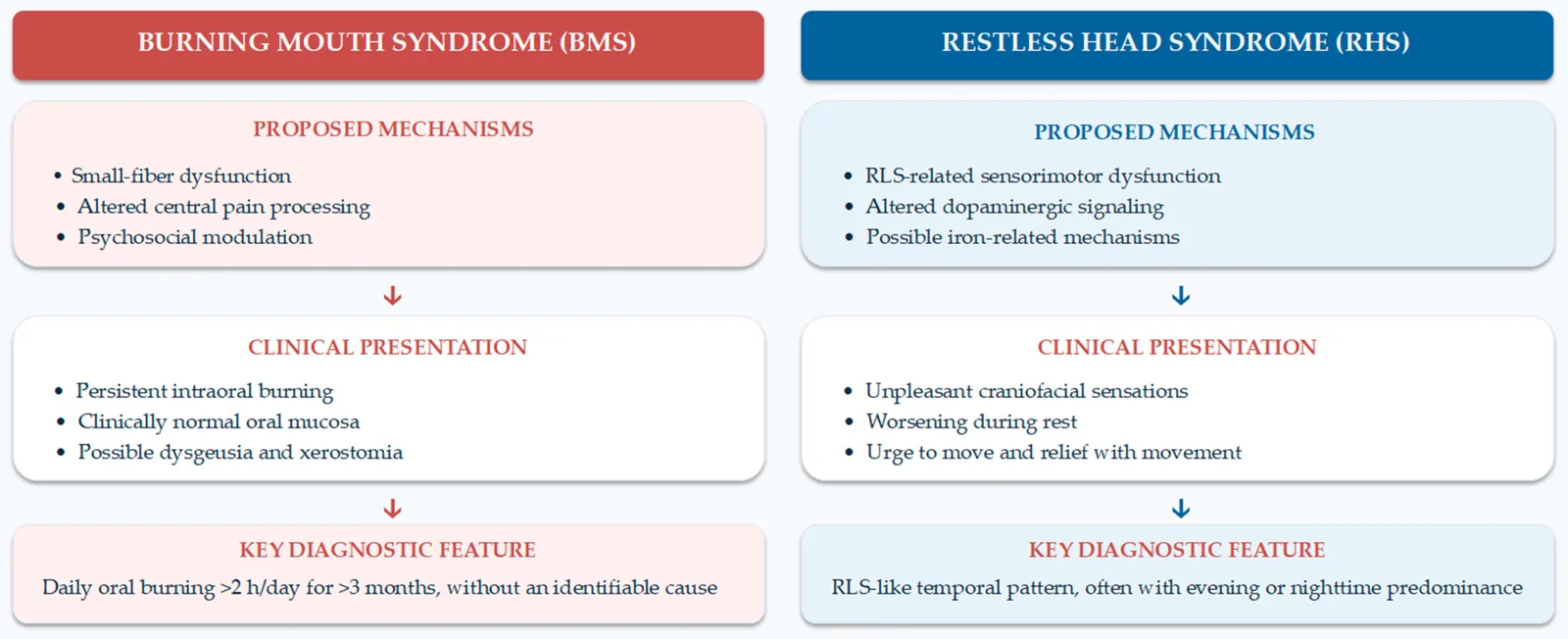
Proposed mechanisms, clinical presentation and key diagnostic features of BMS and RHS.

This table summarizes published case reports and case series describing RHS, BMS, and variants of RLS with involvement of the craniofacial or oral region. The table includes clinical presentation, diagnostic context, and reported treatment outcomes. * Published before 2000; identified through backward citation searching. Group A provides only indirect evidence regarding BMS; Group B is the only direct evidence of clinical overlap between an oral RLS variant and BMS; Group C is shown for comparison of the symptom pattern. Figure 2 shows the mechanisms and clinical characterization of BMS and RHS.

## 3. Results

The search identified 778 records (515 PubMed and 263 ScienceDirect). After removal of 67 duplicates, 711 titles and abstracts were screened and 271 full texts were assessed for eligibility, of which 229 were excluded. A total of 42 publications were included (Figure 1). RHS was consistently described as a variant of RLS and fulfilled the diagnostic principles of RLS as defined by the IRLSSG. Reported cases demonstrated rest-associated discomfort localized to the head or face, symptom worsening in the evening, and partial or complete response to dopaminergic therapy.

The available evidence consisted predominantly of case reports and small case series.

Several studies described BMS in association with neuropathic pain mechanisms, stress, and psychological comorbidities. Neuropathic pain may result from damage or dysfunction of the somatosensory nervous system, leading to altered sensory processing and abnormal pain perception. Peripheral nerve dysfunction and central sensitization may contribute to spontaneous burning sensations, dysesthesia, and pain in the absence of visible tissue abnormalities. In a subset of patients, particularly those who also met diagnostic criteria for RLS or reported symptom relief with movement, BMS symptoms responded to dopaminergic agents. This observation, however, derives from a single case series of five patients, four of whom fulfilled RLS criteria [24]. Table 1 summarizes the clinical characteristics and treatment outcomes documented in case reports and case series on RHS, BMS, and craniofacial variants of RLS.

## 4. Discussion

RHS and BMS are two neuropsychiatric disorders that affect patients in their quality of life [32]. In both conditions, patients suffer from chronic discomfort, pain or burning symptoms. Due to their unspecific symptoms these diseases are often underdiagnosed. RHS is described as a restlessness or discomfort of the head and neck area comparable to RLS. It can occur isolated or in combination with RLS or RAS [19]. Clinically, RHS may resemble migraine, tension-type headache or TMJ disorders, making differential diagnosis challenging [33]. Suzuki et al. (2021) further demonstrated an association between RLS and central sensitization [33].

Due to symptom overlap with common headache and musculoskeletal conditions, patients are often referred to neurologists and orthopedists, and the underlying disorder may remain unrecognized. In some cases, RHS may also be misdiagnosed as a psychiatric disorder.

Headache accompanying orofacial pain may have different origins, including primary headache disorders such as migraine or tension-type headache, temporomandibular disorders (TMD), cervical and masticatory myofascial pain, or neuropathic conditions. Referred pain and overlapping trigeminal and cervical nociceptive pathways may contribute to the clinical overlap. Consequently, headache alone does not establish a diagnosis of RHS, and the relationship to rest, movement, sleep and jaw function should be assessed [34].

### 4.1. Potential Link Between RHS and BMS

BMS is characterized by a burning sensation in the mouth without an apparent cause. Its etiology is multifactorial and may be triggered by hormonal imbalances, neuropathic dysfunction, nutritional deficiencies, and psychological stress [35]. Symptoms such as pain, discomfort and dysfunction of the nervous system are present in both diseases. While RHS—similar to RLS—occurs predominantly in the evening, BMS symptoms can manifest at any time [36,37].

Although the symptom profiles of RHS and BMS differ, both are rare conditions marked primarily by sensory disturbances [38]. The pathogenesis of BMS remains poorly understood. Neuropathic mechanisms as well as dryness of the oral cavity are strongly suggested for being a trigger in BMS [39,40]. Other studies suggest that psychiatric disorders such as post-traumatic stress disorder, depression, or anxiety may also play a causative role [30]. Taiminen et al. (2011) found that 19% of the patients with BMS showed personality disorders, all of which preceded the onset of orofacial pain [41]. It has been hypothesized that low brain dopamine activity contributes to chronic pain. Stress and pain could reduce dopamine output in the brain [42]. In summary, it can be suggested that BMS may trigger or exacerbate a psychiatric illness but can also be triggered by a psychiatric illness [43,44].

A clinically notable feature reported in published cases of RHS is responsiveness to dopaminergic treatment. Microstructural changes in the basal ganglia have been proposed as an underlying cause of the symptoms observed in RLS and its variants [45].

While RHS and BMS are distinct in their clinical manifestations, it has been hypothesized that they share neurophysiological features [24]. Both involve sensory disturbances that are poorly understood and often considered to be of central nervous system origin [9]. It has been proposed that dysfunction in the central or peripheral nervous systems, such as altered pain processing, nerve damage, or changes in neurotransmitter levels, could contribute to the development of both RHS and BMS [9]. Both syndromes are also associated with stress, sleep disturbances and psychiatric comorbidity. However, these features are non-specific and common to many chronic pain conditions; like the similarity of the clinical manifestations, their co-occurrence does not constitute evidence of a common pathophysiological mechanism. At present, the hypothesis of a shared mechanism rests on phenomenological similarities and on the therapeutic response of a small number of patients, and has not been tested in mechanistic or controlled studies.

Whether patients with RHS are at increased risk of developing BMS, or vice versa, is unknown. This remains a hypothesis that requires investigation in epidemiological studies, although it is plausible given the high levels of anxiety, depression, and sleep disruption associated with both conditions [46]. The similarity in symptoms, such as burning or discomfort in the head, neck, and oral regions, indicates, at minimum, that they share common diagnostic pitfalls, making it difficult for clinicians to accurately identify and treat these disorders.

### 4.2. Differential Diagnosis of Craniofacial RLS Variants and BMS

Because no biomarker distinguishes these conditions, the differential diagnosis depends on a careful history with specific attention to the relationship of symptoms to rest, movement and time of day (Table 2). In craniofacial RLS variants, symptoms are accompanied by an urge to move the affected region, begin or worsen during rest or inactivity, are at least partially and temporarily relieved by movement (e.g., moving the head, jaw or tongue), and follow a circadian pattern with evening and night-time predominance, often causing difficulty falling asleep [11,17,22,25]. In primary BMS, by contrast, the defining symptom is an intraoral burning sensation without an urge to move; symptoms are typically absent or mild on waking and increase during the day or are constant, are not relieved by movement, and are often temporarily reduced by eating or drinking [5,28,29]. Concomitant limb symptoms, a family history of RLS, iron deficiency, renal failure, pregnancy or medications known to provoke RLS (e.g., antidepressants and neuroleptics) should prompt consideration of an RLS variant [11,15]. Where uncertainty remains, a structured RLS interview, measurement of serum ferritin and, if available, polysomnography or actigraphy to detect periodic limb movements may support the diagnosis, although periodic limb movements are not specific for RLS [11]. In all cases, local and systemic causes of secondary BMS must be excluded before either diagnosis is made [5,27].

### 4.3. Therapeutic Considerations

Several case reports suggest that dopaminergic therapy may be effective in managing orofacial manifestations resembling RLS, consistent with the hypothesis that these symptoms represent variants of the disorder. Suzuki et al. (2017) described a patient presenting with abnormal facial sensations who fulfilled all diagnostic criteria for RLS [22]. The authors proposed the term “restless face” for this variant, and treatment with pramipexole at a dose of 0.125 mg/day resulted in complete resolution of symptoms [22].

Similarly, Fukunishi et al. (1998) reported a case involving a patient undergoing hemodialysis for end-stage renal disease who experienced facial paresthesias with RLS-like characteristics—symptoms worsening at rest, particularly in the evenings, and relieved by movement [23]. Treatment with levodopa proved effective in alleviating the symptoms [23].

Further support for the dopaminergic responsiveness of orofacial RLS variants comes from Jung et al. (2017), who documented cases of patients experiencing oral discomfort when the mouth was in a relaxed position, accompanied by an urge to move the jaw for temporary relief [25]. These symptoms markedly improved with pramipexole therapy. In another case series, Prakash et al. (2012) described patients suffering from burning sensations throughout the oral cavity who responded favorably to levodopa/carbidopa [24]. Notably, symptom recurrence was observed upon dose reduction or discontinuation, suggesting a dopaminergic contribution in these particular patients [24].

Collectively, these cases suggest that RLS-like symptoms in the orofacial region—though rare—may respond to pharmacological agents typically used for classical RLS, particularly dopamine agonists and levodopa preparations. These findings highlight the importance of considering RLS variants in the differential diagnosis of unexplained orofacial sensory symptoms and the potential benefit of dopaminergic therapy in selected cases. However, all of these observations derive from uncontrolled single cases or very small case series without blinding or placebo control and are subject to publication bias; they cannot establish treatment efficacy.

When symptoms resemble BMS, clinicians should first evaluate local and systemic contributors, including mucosal disease, candidiasis, medication-related dryness, nutritional deficiencies and salivary dysfunction. Saliva substitutes and other moisturizing products may improve xerostomic discomfort but have not consistently reduced primary BMS pain. Depending on the clinical assessment, topical clonazepam, selected neuropathic pain medications and cognitive behavioral interventions may be discussed, acknowledging the limited and heterogeneous evidence and potential adverse effects [47,48]. These approaches differ from the assessment and management of suspected craniofacial RLS variants, for which specialist neurological evaluation is important.

Treatment should be directed at the underlying diagnosis rather than at burning or headache symptoms alone. For patients with myofascial pain or TMD, initial conservative measures include patient education, avoidance of parafunctional loading, jaw exercises, physiotherapy, and, where appropriate, a reversible occlusal splint. Splints should not be regarded as a treatment for RHS or primary BMS; a systematic review found insufficient evidence that they are superior to other interventions for orofacial myalgia [49]. Biofeedback-based approaches may also be considered for selected patients with excessive masticatory muscle activity, although the evidence remains heterogeneous [50]. Analgesics or other pharmacological treatments may be considered according to the specific headache or TMD diagnosis. Botulinum toxin has been investigated for selected refractory orofacial pain conditions, but its indications, comparative efficacy and treatment protocols remain uncertain; it should not be presented as routine first-line treatment [51].

Therapeutic approaches for primary BMS vary widely, as no standardized treatment protocol currently exists. Pharmacological options include capsaicin, clonazepam, duloxetine, amitriptyline, gabapentin, and pregabalin [27]. In addition, non-pharmacological interventions such as laser therapy, transcranial magnetic stimulation, psychotherapy, botulinum toxin injections, and electroconvulsive therapy have also been explored [27]. In cases of secondary BMS, treatment is primarily directed at addressing the underlying cause. Prakash et al. (2012) reported in a case series involving five patients that all showed a positive response to levodopa; notably, four of these patients also met the diagnostic criteria for RLS [24].

These observations suggest that dopaminergic mechanisms may be involved in a subset of patients whose oral symptoms fulfil RLS criteria. They neither establish a shared aetiology of RHS and BMS nor demonstrate the efficacy of dopaminergic treatment in typical primary BMS, for which there is no supporting evidence. It is therefore essential to distinguish BMS accompanied by RLS features, which may represent a misclassified oral RLS variant, from primary BMS, for which established BMS treatments remain first-line [27].

The limitations of dopaminergic therapy must also be considered. Levodopa and dopamine agonists may cause nausea, orthostatic hypotension, daytime somnolence and impulse control disorders [52,53]. The most relevant long-term complication in RLS is augmentation, an iatrogenic worsening of symptoms characterized by earlier onset during the day, increased intensity, shorter latency at rest and spread to previously unaffected body regions [15,52]. Augmentation is most frequent with levodopa but also develops during long-term treatment with dopamine agonists, and its risk increases with dose and duration of treatment [15,52]. Because anatomical spread of symptoms to the arms, trunk or face is a recognized feature of augmentation, new craniofacial symptoms in a patient already receiving dopaminergic treatment should prompt consideration of augmentation rather than of a separate condition [15,52]. Reflecting these concerns, the current American Academy of Sleep Medicine guideline recommends against the standard use of levodopa and dopamine agonists for RLS and favours iron supplementation in patients with low iron stores and alpha-2-delta ligands (gabapentin, gabapentin enacarbil, pregabalin) as first-line options [53]. Notably, alpha-2-delta ligands are also used in the treatment of BMS [27]. Any dopaminergic trial in patients with a suspected craniofacial RLS variant should therefore be low-dose, time-limited and closely monitored, and such treatment is not justified in primary BMS.

### 4.4. Underdiagnosis and Misdiagnosis

One of the primary reasons for the underdiagnosis of RHS and BMS is the lack of awareness among healthcare providers. Both conditions are often mistaken for other more common disorders, leading to delays in diagnosis and inadequate treatment [46]. In particular, BMS is frequently misattributed to oral infections, dental issues, or allergies, while RHS is often confused with temporomandibular joint dysfunction, tension headaches, or migraines. As both conditions lack definitive diagnostic tests, clinicians may rely on symptom history and exclusion of other causes, which can lead to misdiagnosis.

A practical referral pathway may begin with a dentist, oral medicine clinician or orofacial pain/TMJ specialist to exclude odontogenic disease, mucosal pathology and TMD. Patients with headache, sensory abnormalities, an urge to move or symptoms predominantly occurring at rest or in the evening should be referred to a neurologist, ideally with expertise in movement or sleep disorders. Persistent BMS-like symptoms may warrant coordinated care with oral medicine and pain specialists; psychological or psychiatric assessment can be helpful when anxiety, depression, insomnia or substantial pain-related distress coexist, without implying that the symptoms are psychogenic. Red flags such as a new severe headache, focal neurological deficits or progressive sensory loss require prompt medical evaluation.

Another factor contributing to the underdiagnosis is the stigma surrounding these conditions. Patients with BMS and RHS often face skepticism, as their symptoms are subjective and there are no obvious physical markers to support the diagnosis. This can lead to frustration and feelings of isolation, further complicating the clinical picture. In some cases, the psychological impact of living with chronic, unexplained discomfort can result in a diagnosis of a psychosomatic or mood disorder, which may not address the underlying cause of the symptoms [46].

To improve the diagnosis and treatment of RHS and BMS, there is a critical need for increased awareness among healthcare professionals. This includes educating clinicians about the unique presentations of these conditions and the potential overlap with other disorders. Improved diagnostic criteria, standardized screening tools, and more research into the pathophysiology of RHS and BMS will help to identify these conditions earlier and lead to more effective treatment options.

Additionally, a multidisciplinary approach involving neurologists, dentists, psychologists, and pain specialists may provide better outcomes for patients with these conditions. Managing BMS and RHS often requires a combination of pharmacological treatments (such as neuropathic pain medications, antidepressants, or anticonvulsants), behavioral therapy, lifestyle modifications, and stress management techniques [27].

The main contribution of this review is the systematic comparison of the clinical characteristics of craniofacial RLS variants and BMS, two conditions that may present with similar sensory complaints but require different diagnostic approaches. By bringing together the available case reports and clinical studies, we highlight the importance of symptom timing, the urge to move, and relief through movement as potential distinguishing features. Furthermore, our findings suggest that some patients presenting with burning oral sensations may have an atypical manifestation of RLS rather than primary BMS. Recognizing these clinical differences may help avoid misdiagnosis and unnecessary treatment. However, the available evidence remains limited, and a direct pathophysiological relationship between these conditions cannot be established.

### 4.5. Limitations

This review has several limitations. First, the available evidence consists almost entirely of single case reports and small case series without control groups, randomization, or blinding, and is therefore subject to substantial selection and publication bias, as favourable treatment responses are more likely to be reported than negative ones. Second, no validated diagnostic criteria exist for RHS or for craniofacial variants of RLS. Third, the proposed link between RHS and BMS rests on phenomenological and therapeutic parallels rather than direct mechanistic evidence, and a shared response to dopaminergic agents does not by itself establish a common pathophysiology, given the broad role of dopamine in pain modulation. Fourth, owing to the inconsistent terminology for craniofacial RLS variants, relevant reports may have been missed despite supplementary citation searching. These limitations reflect the early state of the field and underscore the need for prospective and controlled studies before firm diagnostic and therapeutic conclusions can be drawn.

## 5. Conclusions

Overall, it can be said that RHS is a very rare and pathophysiological poorly understood disease. RHS and BMS represent two neuropsychiatric conditions that are often underdiagnosed and misdiagnosed, leading to unnecessary suffering for patients. The currently available evidence, which consists predominantly of case reports and small case series, supports clinical similarities between craniofacial RLS variants and BMS and justifies considering atypical RLS variants in the differential diagnosis of unexplained orofacial sensory complaints. It does not, however, confirm a direct pathophysiological association between RHS and BMS, and the response to dopaminergic agents observed in individual patients does not establish their efficacy in primary BMS. Future research should prioritize (i) the development and validation of standardized diagnostic criteria for craniofacial RLS variants; (ii) multicenter cross-sectional studies that systematically screen patients with BMS for RLS features, and patients with RLS for orofacial symptoms, to determine the true prevalence of co-occurrence; and (iii) prospective, ideally randomized and placebo-controlled, studies of treatment response, including non-dopaminergic options such as alpha-2-delta ligands and iron, with long-term monitoring for augmentation. Such studies are required to determine whether the observed clinical similarities reflect a genuine pathophysiological association or merely overlapping symptom profiles.

By increasing awareness, improving diagnostic approaches, and investing in further research, we can better address these conditions, improve patient outcomes, and reduce the stigma associated with them. Clarifying whether a true connection between RHS and BMS exists could open up new avenues for research and treatment, ultimately improving the lives of those affected by these underrecognized conditions.

## Figures and Tables

**Table 2:** Clinical features that may help to distinguish craniofacial RLS variants from primary BMS.

Feature	Craniofacial RLS Variants (RHS, Restless Face, Restless Mouth)	Primary BMS
**Core symptom**	Unpleasant sensation with an urge to move the head, face, jaw or tongue	Intraoral burning or dysaesthesia; no urge to move
**Relationship to rest**	Begins or worsens during rest or inactivity	No consistent relationship to rest
**Effect of movement**	Partial or complete, temporary relief	No relief by movement; eating or drinking may temporarily relieve symptoms
**Time of day**	Evening and night-time predominance	Often minimal on waking and increasing during the day, or constant
**Sleep**	Difficulty falling asleep caused by the symptoms	Sleep disturbance common, usually secondary to pain or mood
**Typical location**	Head, scalp, neck, face, jaw or tongue; often with limb symptoms	Tongue (tip and lateral borders), lips, anterior palate; usually bilateral
**Associated findings**	Limb RLS, family history of RLS, low ferritin, periodic limb movements	Dysgeusia, subjective xerostomia
**Diagnostic framework**	IRLSSG criteria applied to the craniofacial region; no validated site-specific criteria	ICOP 2020 criteria after exclusion of secondary causes
**Response to dopaminergic agents**	Reported in case reports and small case series	Not established; reported only in patients with RLS features

BMS, burning mouth syndrome; ICOP, International Classification of Orofacial Pain; IRLSSG, International Restless Legs Syndrome Study Group; RHS, restless head syndrome; RLS, restless legs syndrome.

## Data Availability

No new data were created or analyzed in this study. Data sharing is not applicable to this article.
